# Trends in Self-perceived Weight Status, Weight Loss Attempts, and Weight Loss Strategies Among Adults in the United States, 1999-2006

**DOI:** 10.1001/jamanetworkopen.2019.15219

**Published:** 2019-11-13

**Authors:** Liyuan Han, Dingyun You, Fangfang Zeng, Xiaoqi Feng, Thomas Astell-Burt, Shiwei Duan, Lu Qi

**Affiliations:** 1 School of Medicine, Department of Epidemiology, Zhejiang Provincial Key Laboratory of Pathophysiology, Ningbo University, Ningbo, Zhejiang Province, China; 2 School of Public Health and Tropical Medicine, Department of Epidemiology, Tulane University, New Orleans, Louisiana; 3 School of Public Health, Kunming Medical University, Kunming City, Yunnan Province, China; 4 School of Medicine, Department of Epidemiology, Jinan University, Guangzhou, Guangdong Province, China; 5 Population Wellbeing and Environment Research Lab, School of Health and Society, Faculty of Social Sciences, University of Wollongong, Wollongong, New South Wales, Australia; 6 Menzies Centre for Health Policy, University of Sydney, Sydney, New South Wales, Australia; 7 School of Public Health, Peking Union Medical College, The Chinese Academy of Medical Sciences, Beijing, China; 8 Clinical and Translational Epidemiology Unit, Division of Gastroenterology, Massachusetts General Hospital, Boston; 9 Department of Nutrition, Harvard T.H. Chan School of Public Health, Boston, Massachusetts; 10 Channing Division of Network Medicine, Department of Medicine, Brigham and Women’s Hospital, Harvard Medical School, Boston, Massachusetts

## Abstract

**Importance:**

The self-perception of weight and weight loss attempts might promote weight loss and maintenance.

**Objective:**

To examine trends in current measured body mass index (BMI) and weight, self-reported weight, self-perceived weight status, weight loss attempts, and weight loss strategies among adults in the United States.

**Design, Setting, and Participants:**

This national cross-sectional study used data from continuous National Health and Nutrition Examination Survey (NHANES) data sets (1999-2000 to 2015-2016). Participants were US residents older than 20 years. Data were analyzed from January 2018 to December 2018.

**Main Outcomes and Measures:**

Current measured BMI and weight, self-reported weight, self-perceived weight status, weight loss attempts, and applied weight loss strategies. Adjusted, self-reported, prior-year weight was calculated using correction equations that considered age, sex, race/ethnicity, and quartile of self-reported prior-year weight.

**Results:**

Data were collected from 48 026 participants (19 792 [41.2%] aged 40-64 years; 24 255 [50.5%] women; 21 725 [45.2%] white) through 9 surveys from 1999-2000 to 2015-2016. Increasing trends were observed in current measured BMI (difference, 1.20; 95% CI, 0.92-1.47; *P *for trend < .001), current measured weight (difference 2.77 kg; 95% CI, 1.92-3.61 kg; *P *for trend < .001), adjusted, self-reported, prior-year weight (difference, 2.36 kg; 95% CI, 1.52-3.21 kg; *P *for trend < .001), and the difference between measured and adjusted self-reported weight (difference 0.70 kg; 95% CI, 0.34-1.07 kg; *P *for trend < .001). During this period, the proportion of overall participants who had attempted to lose weight increased from 34.3% to 42.2% (difference, 8.0%; 95% CI, 4.1%-10.5%; *P* for trend < .001). The most commonly reported weight loss strategies with the most rapidly increasing prevalence during the study period were reduced food consumption (21.2%-31.9%; difference, 11.1%; 95% CI, 8.2%-13.3%; *P *for trend < .001), exercise (18.2%-31.5%; difference, 14.4%; 95% CI, 11.3%-16.9%; *P *for trend < .001), and frequent water intake (0.2%-26.3%; difference, 26.2%; 95% CI, 24.1%-29.0%; *P *for trend < .001). Between 2005-2006 and 2015-2016, increases were also observed for the reported consumption of more fruits, vegetables, and salads (0.1%-29.4%; difference, 30.3%; 95% CI, 28.1%-31.2%; *P *for trend < .001), changing eating habits (0.3%-20.5%; difference, 20.2%; 95% CI, 19.1%-22.3%; *P *for trend < .001), and the consumption of less sugar, candy, and sweets (0.2%-20.9%; difference, 21.7%; 95% CI, 19.3%-22.6%; *P *for trend < .001).

**Conclusions and Relevance:**

In this cross-sectional study, our data indicated an increasing trend in the proportion of participants who attempted to lose weight and a parallel increasing trend in current measured BMI and weight among adults in the United States.

## Key Points

**Question:**

What were the trends in current measured body mass index and weight, self-perceived weight status, weight loss attempts, and weight loss strategies in adults in the United States from 1999 to 2016?

**Findings:**

In this cross-sectional study with data from 48 026 participants in the National Health and Nutrition Examination Survey, increasing trends were observed in current measured body mass index and weight; adjusted, self-reported, prior-year weight; and the difference between current measured weight and adjusted, self-reported, prior-year weight. The proportion of participants who attempted to lose weight increased during the study period.

**Meaning:**

In this study, an increased trend in the proportion of participants who attempted to lose weight was observed, despite increased trends in current and historical weight.

## Teaser

This cross-sectional study examines trends in current measured body mass index (BMI) and weight, self-reported weight, self-perceived weight status, weight loss attempts, and weight loss strategies among adults in the United States using data from the National Health and Nutrition Examination Survey.

## Introduction

Obesity is associated with a variety of major chronic diseases, including cardiovascular disease, type 2 diabetes, and cancer, as well as premature mortality.^1,2^ Compelling evidence suggests that even moderate (ie, 3%-5%) weight loss significantly reduces the risk of obesity-related diseases and mortality.^3^ However, losing weight and maintaining a healthy weight remain significant challenges.^4^

Previous studies have shown that self-perception of weight and weight loss attempts might promote weight loss and maintenance.^5,6^ In addition, the application of weight loss strategies has been associated with successful weight loss in adults with obesity in the United States.^7^ However, nationwide data regarding trends in current measured body mass index (BMI; calculated as weight in kilograms divided by height in meters squared) and weight, self-reported weight, self-perceived weight status (eg, self-perceived weight and the intention to weigh more, less, or the same amount), weight loss attempts, and weight loss strategies in the adult population in the United States are scarce.

Using nationally representative data from 9 continuous applications of the National Health and Nutrition Examination Survey (NHANES; 1999-2000 to 2015-2016), we aimed to estimate temporal trends in current measured BMI and weight, self-reported weight, self-perceived weight status, and attempts and strategies used for weight loss among adults in the United States. We also examined the trend in adjusted, self-reported, prior-year weight using a corrected equation.^8^ Trends in weight loss strategies among those who had overweight or obesity or perceived themselves as having overweight or obesity were also analyzed. We also focused on the weight differences between the last and current years to determine whether those who attempted to lose weight and used different strategies successfully lost weight.

## Methods

### Study Design and Population

The NHANES is a national series of cross-sectional and multistage probability surveys representative of the noninstitutionalized population of the United States.^9^ Data in NHANES have been collected continuously in 2-year surveys since 1999. We used data from 9 consecutive surveys, covering the period of 1999-2000 to 2015-2016. We restricted our analyses to nonpregnant participants 20 years or older. The NHANES was approved by the National Center for Health Statistics Ethics Review Board. All participants provided informed consent. The Medical School of Ningbo University institutional review board determined that the current study was exempt from review and informed consent given the use of publicly available data. This study followed the Strengthening the Reporting of Observational Studies in Epidemiology (STROBE) reporting guideline for cross-sectional design.

The NHANES collects data through home interviews and physical examinations. During home interviews, participants were asked questions about demographic, socioeconomic, dietary, and health-related parameters. The detailed methodology and protocols are available on the NHANES website.^9^

### Assessment of Self-perceived Weight Status and Weight Loss Attempts and Strategies

A weight history questionnaire was administered to participants 16 years and older during household interviews. This questionnaire solicited personal data about several topics related to body weight, including self-reported weight (ie, self-reported weight over a lifetime), self-perceived weight status (ie, “how do you consider your weight” and whether participants intend to weigh more, less, or the same amount), weight loss attempts during the past year (ie, “during the past 12 months, have you tried to lose weight”), and applied weight loss strategies (eg, reduced food intake).

Self-reported weight was recorded for current weight, prior-year weight, weight 10 years earlier, weight at age 25 years, and highest-ever weight. Measured weight was measured in pounds using electronic scales in the mobile examination center. Self-reported current and previous weights were standardized to weight in pounds using a conversion factor of 2.2046 pounds per kilogram. In this study, current measured weight was converted from pounds to kilograms using the same conversion formula.

Self-perceived weight status was estimated from participant response to the question, “How do you consider your weight?” (possible responses, overweight, underweight, or approximately the right weight) and to the item inquiring whether the participant intends to weigh more, less, or the same amount. Other questions, such as, “During the past 12 months, have you tried to lose weight?” addressed weight loss attempts. Participants whose self-reported current weight was at least 10 pounds lower than their reported prior-year weight were asked whether that weight change had been intentional. Those who answered yes were categorized as having tried to lose weight. All other participants, including those who reported an unintentional weight loss of at least 10 pounds, were asked directly, “During the past 12 months, have you tried to lose weight?”^10^

Participants who reported a weight loss attempt during the previous year were asked to provide further details about applied strategies. From 1999-2000 to 2015-2016, the NHANES provided a list of 14 to 20 options. The following 14 options were included in all surveys: (1) reduced food intake, (2) switched to less caloric foods, (3) reduced fat intake, (4) exercised, (5) skipped meals, (6) consumed diet foods or products, (7) used a liquid diet formula, (8) participated in a weight loss program, (9) used prescription diet pills, (10) used nonprescription diet pills, (11) used laxatives or vomiting, (12) consumed large volumes of water, (13) adhered to a special diet, or (14) other. Starting in 2005, the NHANES survey included 6 additional options, as follows: (1) reduced carbohydrate intake; (2) began or resumed a smoking habit; (3) increased intake of fruits, vegetables, and salads; (4) altered eating habits (eg, no food consumption late at night, several small meals per day); (5) reduced intake of sugar, candy, and sweets; and (6) reduced consumption of junk food or fast food. The strategies were not mutually exclusive.

The above questions were presented to participants 16 years and older. Participants 85 years and older were top-coded at 85 years of age. Data about several demographic parameters were collected, including age (ie, 20-39, 40-64, and ≥65 years), sex (ie, male and female), race/ethnicity (ie, non-Hispanic white, non-Hispanic black, Mexican-American, and other), and BMI (ie, normal weight, 18 to <25; overweight, 25 to ≤30; and obesity, ≥30).

### Statistical Analysis

Prevalence estimates and SEs were calculated using the procedure proc surveymeans. Calculations with SEs of 30% or less were considered reliable.^11^ If the relative SE exceeded 30%, data were suppressed (empty cells), consistent with NHANES reporting guidelines.^12^

For continuous variables, linear trends across each survey were calculated using the procedure proc surveyreg after adjusting for age, sex, and race/ethnicity. For categorical variables, trends across each survey were calculated by including the midpoint of each survey as a continuous variable in the logistic regression after adjusting for age, sex, and race/ethnicity. A Taylor series linearization was used to estimate variance.^11^

Odds ratios (ORs) and 95% CIs were calculated using the procedure proc surveylogistic, and the prevalence ratios are presented. Increasing trends were defined as differences greater than zero or ratios greater than 1, with *P *for trend ≤ .001; decreasing trends were defined as differences less than 0 or ratios less than 1, with *P *for trend ≤ .001; and stable trends were defined as *P *for trend > .001.

To reduce type I error induced by multiple tests, Bonferroni correction was applied; *P* ≤ .001 was adopted as the threshold for statistical significance, and all tests were 2-tailed. All statistical analyses were performed using SAS statistical software version 9.4 (SAS Institute) and designed to account for the complex weighted sampling design of the NHANES.^9^

## Results

Data were collected from 48 026 participants (19 792 [41.2%] aged 40-64 years; 24 255 [50.5%] women; 21 725 [45.2%] white) through 9 surveys from 1999-2000 to 2015-2016. The characteristics of the participants are summarized in the eFigure and eTable 1 in the Supplement. The sample sizes stratified by age, sex, race/ethnicity, and BMI are also shown in eTable 1 in the Supplement. Participants’ weight 10 years earlier, weight at age 25 years, highest-ever weight, and age of highest-ever weight appear in eTable 2 in the Supplement.

From 1999-2000 to 2015-2016, significant increases in current measured BMI (difference, 1.20; 95% CI, 0.92-1.47; *P *for trend < .001), current measured weight (difference 2.77 kg; 1.92-3.61 kg; *P *for trend < .001), adjusted, self-reported, prior-year weight (difference, 2.36 kg; 95% CI, 1.52-3.21 kg; *P *for trend < .001), and the difference between current measured weight and adjusted, self-reported, prior-year weight (difference 0.70 kg; 95% CI, 0.34-1.07 kg; *P *for trend < .001) were observed (Table 1). Among those who attempted to lose weight, increased trends were observed for current measured BMI (difference, 1.21; 95% CI, 0.69-1.73; *P *for trend < .001), current measured weight (difference, 2.55 kg; 95% CI, 0.93-4.17 kg; *P *for trend < .001), and the difference between current measured weight and unadjusted, self-reported, prior-year weight (difference 0.55 kg; 95% CI, 0.37-0.86 kg; *P *for trend < .001) (Table 1). Among those who did not attempt to lose weight, an increasing trend was observed for the difference between current measured weight and adjusted, self-reported, prior-year weight (difference, 0.85 kg; 95% CI, 0.45-1.25 kg; *P *for trend < .001) (Table 1). The weighted prevalence of overall participants who attempted to lose weight during the previous year increased from 34.3% in 1999-2000 to 42.2% in 2015-2016 (difference, 8.0%; 95% CI, 4.1%-10.5%, *P *for trend < .001) (Table 2).

**Table 1.** Trends in Current Measured BMI and Weight; Adjusted, Prior-Year, Self-reported Weight; Weight Loss Attempts; and Weight Loss Strategies Among Adults in the United States, 1999-2000 to 2015-2016^a^

Characteristic	1999-2000 (n = 4597)	2001-2002 (n = 5094)	2003-2004 (n = 4808)	2005-2006 (n = 4643)	2007-2008 (n = 5878)	2009-2010 (n = 6150)	2011-2012 (n = 5503)	2013-2014 (n = 5704)	2015-2016 (n = 5649)	*P* for Trend^b^	2015-2016 vs 1999-2000, Difference (95% CI)
**All Participants**											
Current measured BMI, No. (%)	4117 (89.6)	4413 (86.6)	4431 (92.2)	4356 (93.8)	5550 (94.4)	5926 (96.4)	5181 (94.1)	5455 (95.6)	5337 (94.5)	NA	NA
Mean (95% CI)	27.96 (27.47 to 28.4)	28.01 (27.68 to 28.33)	28.20 (27.87 to 28.50)	28.54 (28.05 to 29.03)	28.54 (28.21 to 28.80)	28.71 (28.45 to 28.90)	28.71 (28.27 to 29.16)	29.16 (28.80 to 29.53)	29.39 (28.84 to 29.90)	<.001	1.20 (0.92 to 1.47)
Current measured weight, No. (%)	4130 (89.4)	4508 (88.5)	4448 (92.5)	4371 (94.1)	5560 (94.6)	5935 (96.5)	5193 (94.4)	5468 (95.9)	5341 (94.5)	NA	NA
Mean (95% CI), kg	79.88 (78.50 to 81.27)	80.30 (79.32 to 81.28)	80.98 (80.00 to 81.90)	81.77 (80.27 to 83.27)	81.68 (80.53 to 82.80)	82.05 (81.20 to 82.90)	81.82 (80.73 to 82.72)	83.02 (82.11 to 83.93)	83.34 (81.75 to 84.90)	<.001	2.77 (1.92 to 3.61)
Adjusted, self-reported, prior-year weight, No. (%)	4062 (88.4)	4656 (91.4)	4464 (92.8)	4353 (93.8)	5507 (93.7)	5843 (95.0)	5149 (93.6)	5414 (94.9)	5299 (93.8)	NA	NA
Mean (95% CI), kg	80.35 (79.09 to 81.60)	80.58 (79.50 to 81.66)	81.54 (80.49 to 82.50)	82.20 (80.89 to 83.51)	81.99 (80.85 to 83.10)	82.15 (81.22 to 83.00)	82.20 (80.98 to 83.41)	82.88 (81.73 to 84.03)	83.33 (81.80 to 84.80)	<.001	2.36 (1.52 to 3.21)
Difference between current and self-reported prior-year weight, No. (%)	4013 (87.3)	4444 (87.2)	4392 (91.3)	4280 (92.2)	5420 (92.2)	5789 (94.1)	5088 (92.5)	5360 (94.0)	5238 (92.7)	NA	NA
Unadjusted self-reported weight, mean (95% CI), kg	1.14 (0.74 to 1.53)	0.76 (0.49 to 1.03)	0.55 (0.13 to 0.97)	0.54 (0.08 to 1.01)	0.65 (0.33 to 0.97)	0.70 (0.44 to 0.97)	0.26 (–0.09 to 0.61)	0.67 (0.18 to 1.15)	0.53 (0.07 to 1.00)	.17	–0.29 (–0.65 to 0.07)
Adjusted self-reported weight, mean (95% CI), kg	–0.44 (–0.85 to –0.04)	–0.44 (–0.73 to –0.14)	–0.65 (–1.11 to –0.18)	–0.36 (–0.85 to –0.14)	–0.31 (–0.66 to –0.04)	–0.11 (–0.36 to 0.15)	–0.50 (–0.89 to –0.12)	0.07 (–0.44 to 0.57)	–0.01 (–0.47 to 0.44)	<.001	0.70 (0.34 to 1.07)
**Participants Who Tried to Lose Weight**											
Current measured BMI, No. (%)	1165 (25.3)	1246 (24.5)	1250 (26.0)	1396 (30.1)	1614 (27.5)	1685 (27.4)	1546 (28.1)	1747 (30.6)	1819 (32.2)	NA	NA
Mean (95% CI)	30.49 (29.62 to 31.36)	30.22 (19.71 to 30.73)	30.25 (29.65 to 30.85)	30.49 (29.75 to 31.23)	30.66 (30.14 to 31.18)	31.02 (30.69 to 31.35)	31.14 (30.46 to 31.82)	31.33 (30.74 to 31.92)	31.89 (31.34 to 32.45)	<.001	1.21 (0.69 to 1.73)
Current measured weight, No. (%)	1168 (25.4)	1261 (24.8)	1253 (26.1)	1397 (30.1)	1616 (27.5)	1686 (27.4)	1548 (28.1)	1752 (30.7)	1823 (32.3)	NA	NA
Mean (95% CI), kg	86.02 (83.49 to 88.55)	85.5 (83.93 to 87.07)	85.44 (83.72 to 87.17)	85.93 (83.49 to 88.37)	86.83 (85.08 to 88.58)	87.57 (86.13 to 89.00)	87.67 (85.90 to 89.44)	87.92 (86.76 to 89.08)	89.71 (88.23 to 91.20)	<.001	2.55 (0.93 to 4.17)
Self-reported prior-year weight, No. (%)	1151 (25.0)	1285 (25.2)	1254 (26.1)	1391 (30.0)	1610 (27.5)	1652 (26.9)	1537 (27.9)	1733 (30.4)	1804 (31.9)	NA	NA
Unadjusted, mean (95% CI), kg	81.72 (79.72 to 83.72)	81.40 (79.75 to 83.05)	81.99 (80.37 to 83.61)	82.30 (80.19 to 84.41)	83.37 (81.63 to 85.10)	83.92 (82.33 to 85.52)	83.74 (82.20 to 85.28)	83.42 (82.05 to 84.80)	85.39 (83.95 to 86.84)	<.001	2.29 (0.75 to 3.82)
Adjusted, mean (95% CI), kg	83.60 (81.55 to 85.65)	82.99 (81.26 to 84.72)	83.53 (81.88 to 85.18)	83.64 (81.51 to 85.78)	84.71 (82.90 to 86.53)	85.09 (83.48 to 86.70)	84.84 (83.25 to 86.42)	84.14 (82.72 to 85.56)	86.13 (84.70 to 87.56)	.02	1.16 (0.39 to 2.72)
Difference between current and self-reported prior-year weight, No. (%)	1143 (24.8)	1242 (24.4)	1237 (25.7)	1373 (29.6)	1588 (27.0)	1588 (25.8)	1644 (29.9)	1520 (26.6)	1723 (30.5)	NA	NA
Unadjusted self-reported weight, mean (95% CI), kg	3.43 (2.95 to 3.91)	4.25 (3.45 to 5.05)	3.94 (3.45 to 4.44)	3.35 (2.87 to 3.83)	3.56 (3.06 to 4.06)	3.54 (3.04 to 4.05)	3.39 (2.88 to 3.9)	3.59 (3.08 to 4.12)	4.35 (3.83 to 4.88)	<.001	0.55 (0.37 to 0.86)
Adjusted self-reported weight, mean (95% CI), kg	2.38 (1.06 to 3.16)	2.36 (1.91 to 2.81)	1.82 (1.35 to 2.28)	2.21 (1.60 to 2.80)	2.19 (1.65 to 2.74)	2.20 (1.65 to 2.74)	2.22 (1.74 to 2.70)	2.52 (1.93 to 3.10)	3.63 (3.07 to 4.19)	.43	1.38 (0.78 to 2.01)
**Participants Who Did Not Try to Lose Weight**											
Current measured BMI, No. (%)	2540 (55.3)	2733 (53.7)	2696 (56.1)	2455 (52.9)	3242 (55.2)	3540 (57.6)	2998 (54.5)	3005 (52.7)	2796 (49.5)	NA	NA
Mean (95% CI)	26.37 (25.83 to 26.90)	26.42 (26.13 to 26.71)	26.53 (26.24 to 26.82)	26.54 (26.14 to 26.94)	26.79 (26.42 to 27.15)	26.98 (26.55 to 27.40)	26.72 (26.17 to 27.27)	27.01 (26.64 to 27.38)	26.95 (26.24 to 27.67)	.02	0.20 (–0.11 to 0.51)
Current measured weight, No. (%)	2548 (55.4)	2804 (55.0)	2709 (56.3)	2466 (53.1)	3250 (52.3)	3547 (57.7)	3007 (54.6)	3013 (52.8)	2796 (49.5)	NA	NA
Mean (95% CI), kg	75.85 (74.13 to 77.57)	76.18 (75.32 to 77.04)	76.94 (76.13 to 77.75)	77.00 (75.90 to 78.11)	77.14 (75.81 to 78.48)	77.45 (76.28 to 78.63)	76.51 (74.91 to 78.11)	77.31 (76.30 to 78.33)	76.82 (74.69 to 78.96)	.93	0.09 (–0.89 to 1.08)
Self-reported prior-year weight, No. (%)	2502 (54.4)	2913 (57.2)	2723 (56.6)	2441 (52.6)	3233 (55.0)	3492 (56.8)	2970 (54.0)	2975 (52.2)	2767 (49.0)	NA	NA
Unadjusted, mean (95% CI), kg	74.53 (72.99 to 76.06)	75.07 (74.26 to 75.89)	75.76 (74.98 to 76.54)	76.44 (75.44 to 77.45)	75.99 (74.77 to 77.21)	76.25 (75.24 to 77.26)	75.66 (74.19 to 77.13)	76.27 (75.38 to 77.16)	75.91 (73.96 to 77.86)	.05	0.82 (–0.10 to 1.73)
Adjusted, mean (95% CI), kg	75.88 (74.28 to 77.78)	75.95 (75.07 to 76.83)	76.62 (75.76 to 77.48)	76.86 (75.81 to 77.91)	76.64 (75.36 to 77.93)	76.73 (75.69 to 77.77)	76.11 (74.60 to 77.62)	76.63 (75.72 to 77.55)	76.15 (74.24 to 78.06)	.81	–0.18 (–1.12 to 0.76)
Difference between current and self-reported prior-year weight, No. (%)	1796 (39.1)	2466 (48.4)	2763 (57.5)	2673 (57.6)	2399 (40.8)	3175 (51.6)	3449 (62.7)	2934 (51.4)	2938 (52.0)	NA	NA
Unadjusted self-reported weight, No. (%), kg	0.75 (0.25 to 1.25)	1.32 (0.85 to 1.78)	0.93 (0.57 to 1.31)	1.10 (0.67 to 1.53)	0.60 (0.17 to 1.03)	1.03 (0.65 to 1.41)	1.24 (0.91 to 1.58)	0.75 (0.41 to 1.08)	0.52 (0.06 to 1.28)	.41	0.20 (0.19 to 0.59)
Adjusted self-reported weight, mean (95% CI), kg	1.78 (1.33 to 2.21)	–0.03 (–0.52 to 0.45)	0.04 (–0.36 to 0.46)	0.25 (–0.23 to 0.73)	0.19 (–0.22 to 0.59)	0.37 (–0.02 to 0.77)	0.76 (0.43 to 1.09)	0.30 (–0.02 to 0.61)	0.54 (0.22 to 0.94)	<.001	0.85 (0.45 to 1.25)
**Participants with Normal BMI or Underweight Who Intended to Weigh Less or the Same Amount**											
Difference between current and prior-year self-reported weight, No. (%)	2754 (59.9)	1078 (21.1)	1187 (24.7)	1171 (25.2)	1086 (18.5)	1371 (22.3)	1425 (25.9)	1458 (25.6)	1429 (25.3)	NA	NA
Unadjusted self-reported weight, mean (95% CI), kg	–1.43 (–2.06 to –0.81)	–1.33 (–1.68 to –0.98)	–1.34 (–1.73 to –0.95)	–1.20 (–1.71 to –0.70)	–1.48 (–1.88 to –1.08)	–1.19 (–1.53 to –0.85)	–1.23 (–1.67 to –0.80)	–1.36 (–1.84 to –0.87)	–1.36 (–1.74 to –0.98)	.18	0.44 (0.32 to 0.61)
Adjusted self-reported weight, mean (95% CI), kg	0.38 (–0.06 to 0.83)	–2.15 (–2.59 to –1.70)	–1.70 (–2.07 to –1.33)	–1.61 (–2.20 to –1.02)	–1.62 (–2.03 to –1.21)	–1.56 (–1.96 to –1.16)	–1.42 (–1.88 to –0.96)	–1.53 (–2.05 to –1.00)	–1.46 (–1.80 to –1.12)	.02	0.61 (0.11 to 1.11)
**Participants with Normal BMI or Underweight Who Intended to Weigh More**											
Difference between current weight and self-reported prior-year weight, No. (%)	1309 (28.5)	225 (4.4)	235 (4.9)	226 (4.9_	218 (3.7)	198 (3.2)	214 (3.9)	184 (3.2)	192 (3.4)	NA	NA
Unadjusted self-reported weight, mean (95% CI), kg	0.46 (–1.37 to 2.29)	1.40 (0.83 to 1.98)	0.94 (0.14 to 1.74)	–0.09 (–0.73 to 0.56)	1.02 (0.20 to 1.85)	0.14 (–0.89 to 1.17)	1.58 (0.98 to 2.18)	0.44 (–0.93 to 1.81)	0.79 (–0.24 to 1.82)	.76	0.40 (0.01 to 1.10)
Adjusted self-reported weight, mean (95% CI), kg	–1.03 (–1.51 to –0.56)	0.26 (–0.27 to 0.79)	–0.08 (–0.99 to 0.82)	–1.34 (–2.10 to –0.57)	–0.09 (–1.09 to 0.91)	–0.97 (–2.04 to 0.09)	0.56 (–0.14 to 1.17)	–0.40 (–1.86 to 1.05)	0.11 (–1.19 to 1.41)	.96	1.34 (0.83 to 1.51)
**Participants With Overweight Who Intended to Weigh Less or the Same Amount**											
Difference between current and self-reported prior-year weight, No. (%)	131 (2.8)	791 (15.5)	819 (17.0)	849 (18.3)	810 (13.8)	1119 (18.2)	1182 (21.5)	1080 (18.9)	1073 (19.0)	NA	NA
Unadjusted self-reported weight, mean (95% CI), kg	–0.93 (–1.52 to –0.29)	–0.01 (–0.86 to 0.86)	–0.26 (–0.85 to 0.31)	–0.79 (–1.28 to –0.31)	–0.48 (–1.11 to 0.15)	–0.39 (–1.06 to 0.28)	0.17 (–0.34 to 0.68)	–0.53 (–1.31 to 0.24)	–0.27 (–0.84 to 0.31)	.32	0.64 (0.41 to 1.10)
Adjusted self-reported weight, mean (95% CI), kg	0.92 (–1.40 to 3.25)	–1.61 (–2.56 to 0.67)	–1.25 (–1.98 to –0.52)	–1.79 (–2.30 to –1.23)	–0.78 (–1.47 to –0.08)	–0.08 (–1.67 to 0.09)	–0.07 (–0.61 to 0.46)	–1.08 (–1.88 to –0.28)	–0.62 (–1.25 to 0.006)	.02	1.20 (0.40 to 1.99)
**Participants With Overweight or Obesity Who Intended to Weigh More**											
Difference between current weight and self-reported prior-year weight, No. (%)	1214 (26.4)	1904 (37.3)	2106 (43.8)	2125 (45.8)	2150 (36.6)	2715 (44.1)	2948 (53.6)	2349 (41.2)	2649 (46.9)	NA	NA
Unadjusted self-reported weight, mean (95% CI), kg	1.02 (0.42 to 1.63)	2.98 (2.28 to 3.68)	2.28 (1.84 to 2.73)	1.98 (1.37 to 2.60)	1.82 (1.29 to 2.35)	2.03 (1.27 to 2.48)	1.80 (1.26 to 2.35)	1.45 (0.92 to 1.99)	1.97 (1.17 to 2.77)	.07	0.51 (0.07 to 1.11)
Adjusted self-reported weight, mean (95% CI), kg	–2.07 (–2.72 to –1.41)	0.88 (0.20 to 1.57)	0.54 (0.08 to 1.00)	0.31 (–0.33 to 0.96)	0.38 (–0.21 to 0.98)	0.61 (0.16 to 1.06)	0.50 (–0.02 to 1.02)	0.28 (–0.30 to 0.85)	1.04 (0.19 to 1.88)	.01	0.59 (0.01 to 1.19)

Abbreviations: BMI, body mass index (calculated as weight in kilograms divided by height in meters squared); NA, not applicable.

^a^ Analyses were restricted to nonpregnant participants 20 years and older who responded to the National Health and Nutrition Examination Survey. Not all participants answered all questions; therefore, the number of participants in different categories were not equal to the total number of participants for the survey cycle. Increasing trends were defined as differences greater than 0 or a ratio greater than 1 with *P* for trend ≤ .001; decreasing trends were defined as differences less than 0 or a ratio of less than 1, with a *P* for trend ≤ .001; stable trends were defined as *P* for trend > .001.

^b^ Adjusted for age, sex, and race/ethnicity.

**Table 2.** Trends in Attempts at Weight Loss and Self-perceived Weight Status Among Adults in the United States, 1999-2000 Through 2015-2016^a^

Characteristic	1999-2000 (n = 4597)	2001-2002 (n = 5094)	2003-2004 (n = 4808)	2005-2006 (n = 4643)	2007-2008 (n = 5878)	2009-2010 (n = 6150)	2011-2012 (n = 5503)	2013-2014 (n = 5704)	2015-2016 (n = 5649)	*P* for Trend^b^	2015-2016 vs 1999-2000^b^	
											Ratio (95% CI)	Difference, % (95% CI)
Tried to lose weight in past year, No. (% [95% CI])	1176 (34.31 [32.23 to 36.38])	1305 (33.72 [32.20 to 35.25])	1271 (35.82 [33.31 to 38.32])	1415 (39.82 [38.05 to 41.59])	1638 (36.33 [34.00 to 38.65])	1694 (34.80 [33.25 to 36.35])	1565 (36.85 [34.29 to 39.41])	1762 (39.42 [37.15 to 41.69])	1817 (42.15 [39.36 to 45.25])	<.001	1.34 (1.21 to 1.46)	8.01 (4.12 to 10.51)
Considered self as having overweight, No. (% [95% CI])	2210 (55.51 [52.94 to 58.08])	2503 (56.61 [54.67 to 58.54])	2417 57.57 [55.31 to 59.83])	2441 (58.57 [55.88 to 61.25])	3007 (55.93 [53.81 to 58.06])	3254 (55.87 [52.91 to 58.84])	2623 (53.46 [50.80 to 56.12])	2926 (55.97 [54.35 to 57.58])	2723 (55.35 [52.27 to 58.47])	.002	0.91 (0.83 to 0.98)	–1.06 (–4.92 to 0.46)
Considered self as having underweight, No. (% [95% CI])	217 (5.33 [4.35 to 6.32])	263 (4.88 [4.12 to 5.65])	254 (4.87 [4.07 to 5.67])	238 (4.63 [3.68 to 5.57])	314 (4.83 [4.08 to 5.58])	293 (4.20 [3.44 to 4.96])	301 (4.77 [3.75 to 5.79])	299 (4.87 [4.27 to 5.48])	260 (3.13 [3.01 to 4.78])	.37	0.85 (0.71 to 1.02)	–2.04 (–2.19 to 0.08)
Considered self about the right weight, No. (% [95% CI])	1743 (39.14 [36.58 to 41.70])	1954 (38.50 [36.46 to 40.53])	1843 (37.55 [35.15 to 39.94])	1762 (36.79 [34.38 to 39.20])	2285 (39.23 [36.87 to 41.59])	2427 (39.91 [36.92 to 42.90])	2325 (41.75 [39.41 to 44.10])	2287 (39.15 [37.59 to 40.70])	2266 (41.25 [38.57 to 44.25])	<.001	1.08 (0.98 to 1.19)	2.11 (0.34 to 4.82)
Intended to weigh more, No. (% [95% CI])	264 (6.79 [5.45 to 8.14])	327 (6.31 [5.43 to 7.19])	320 (6.22 [5.35 to 7.10])	336 (6.67 [5.93 to 7.41])	422 (6.90 [5.90 to 7.91])	423 (5.90 [5.07 to 6.74])	427 (6.50 [5.12 to 7.89])	438 (6.93 [6.11 to 7.76])	407 (6.12 [5.56 to 7.36])	<.001	0.96 (0.82 to 1.12)	–1.02 (–2.02 to 0.83)
Intended to weigh less, No. (% [95% CI])	2434 (62.36 [59.89 to 64.84])	2772 (64.04 [62.28 to 65.81])	2638 (63.42 [61.14 to 65.69])	2721 (66.54 [64.05 to 69.02])	3439 (65.64 [63.84 to 67.43])	3715 (65.13 [62.54 to 67.73])	3081 (64.45 [61.87 to 67.03])	3380 (65.23 [63.17 to 67.29])	3322 (67.24 [64.34 to 70.65])	<.001	1.06 (0.90 to 1.24)	5.01 (1.29 to 6.61)
Intended to weigh about the same, No. (% [95% CI])	1476 (30.83 [28.51 to 33.15])	1627 (29.63 [27.77 to 31.49])	1556 (30.35 [28.19 to 32.50])	1387 (26.78 [24.70 to 28.86])	1752 (27.44 [25.76 to 29.13])	1850 (28.95 [26.56 to 31.33])	1747 (29.03 [26.90 to 31.15])	1698 (27.82 [26.25 to 29.40])	1532 (25.89 [23.12 to 28.23])	<.001	0.83 (0.70 to 0.97)	–6.04 (–7.16 to –0.02)

^a^ Analyses were restricted to nonpregnant participants 20 years and older who responded to the National Health and Nutrition Examination Survey. Not all participants answered all questions; therefore, the number of participants in different categories were not equal to the total number of participants for the survey cycle. Increasing trends were defined as differences greater than 0 or a ratio greater than 1 with *P* for trend ≤ .001; decreasing trends were defined as differences less than 0 or a ratio of less than 1, with a *P* for trend ≤ .001; stable trends were defined as *P* for trend > .001.

^b^ Adjusted for age, sex, and race/ethnicity.

Table 3 presents the trends in weight loss strategies among participants who attempted to lose weight during the previous year. From 1999-2000 to 2015-2016, the most commonly applied strategies were reduced food consumption (eg, 30.8% [95% CI, 29.2%-32.4%] of participants in 2005-2006 and 31.9% [95% CI, 30.1%-33.8%] of participants in 2015-2016), exercise (eg, 29.5% [95% CI, 27.1%-31.8%] of participants in 2005-2006 and 31.5% [95% CI, 28.7%-34.3%] of participants in 2015-2016), and consumption of a large volume of water (eg, 21.6% [95% CI, 19.1%-24.0%] of participants in 2005-2006 and 26.3% [95% CI 23.9%-28.7%] of participants in 2015-2016). The proportion of overall participants who reported reduced food consumption as a weight loss attempt increased from 21.2% to 31.9% during the study period (difference, 11.1%; 95% CI, 8.2%-13.3%; *P *for trend < .001). The proportion of overall participants who reported exercise as a weight loss strategy increased from 18.2% to 31.5% (difference, 14.4%; 95% CI, 11.3%-16.9%, *P *for trend < .001). The proportion of overall participants who reported consumption of a large volume of water increased from 0.2% to 26.3% (difference, 26.2%; 95% CI, 24.1%-29.0%; *P *for trend < .001). From 2005-2006 to 2015-2016, significantly increased trends were also observed in the proportions of overall participants who reported consuming more fruits, vegetables, and salads (0.1%-29.4%; difference, 30.3%; 95% CI, 28.1%-31.2%; *P *for trend < .001); changing their eating habits (0.3%-20.5%; difference, 20.2%; 95% CI, 19.1%-22.3%; *P *for trend < .001); consuming less sugar, candy, and sweets (0.2%-20.9%; difference, 21.7%; 95% CI, 19.3%-22.6%; *P *for trend < .001); or consuming less junk food or fast food (12.8%-24.3%; difference, 12.1%; 95% CI, 10.1%-14.5%; *P *for trend < .001).

**Table 3.** Trends in Weight Loss Strategies Among Adults in the United States 20 Years or Older Who Tried to Lose Weight in the Past Year, 1999-2000 to 2015-2016^a^

Strategy	No. (% [95% CI])									*P* for Trend^b^	2015-2016 vs 1999-2000^b^	
	1990-2000 (n = 4597)	2001-2002 (n = 5094)	2003-2004 (n = 4808)	2005-2006 (n = 4643)	2007-2008 (n = 5878)	2009-2010 (n = 6150)	2011-2012 (n = 5503)	2013-2014 (n = 5704)	2015-2016 (n = 5649)		Ratio^c^	Difference, % (95% CI)
Ate less food	813 (21.18 [19.23 to 23.12])	1163 (26.28 [24.68 to 27.89])	1144 (27.83 [25.50 to 30.15])	1282 (30.79 [29.20 to 32.38])	1549 (29.15 [26.82 to 31.48])	1387 (24.28 [22.83 to 25.73])	1328 (27.88 [25.30 to 30.46])	1496 (29.32 [27.71 to 30.93])	1572 (31.93 [30.08 to 33.77])	<.001	1.59 (1.44 to 1.75)	11.14 (8.23 to 13.34)
Lowered calories	413 (11.77 [9.87 to 13.67])	630 (14.98 [13.78 to 16.18])	673 (17.62 [16.17 to 19.06])	718 (17.83 [16.39 to 19.27])	891 (17.53 [15.96 to 19.09])	664 (12.68 [11.24 to 14.12])	614 (13.59 [11.89 to 15.30])	784 (16.43 [15.09 to 17.78])	857 (17.49 [16.21 to 19.67])	.003	1.53 (1.34 to 1.73)	7.01 (4.45 to 8.56)
Ate less fat	508 (13.09 [11.35 to 14.82])	788 (18.48 [16.62 to 20.34])	712 (17.65 [15.50 to 19.80])	824 (19.81 [18.24 to 21.38])	1077 (20.03 [17.94 to 22.11])	765 (12.68 [11.51 to 13.84])	652 (12.63 [10.87 to 14.39])	719 (13.07 [11.53 to 14.60])	859 (15.37 [13.93 to 16.81])	<.001	1.15 (1.01 to 1.29)	2.28 (1.01 to 3.78)
Exercised	630 (18.22 [16.76 to 19.68])	1003 (24.60 [22.36 to 26.84])	946 (24.67 [22.61 to 26.72])	1152 (29.46 [27.10 to 31.83])	1310 (26.86 [23.94 to 29.78])	1387 (25.79 [24.53 to 27.06])	1351 (27.59 [25.30 to 29.88])	1532 (29.76 [27.81 to 31.71])	1600 (31.52 [28.70 to 34.34])	<.001	2.24 (2.01 to 2.48)	14.36 (11.29 to 16.89)
Skipped meals	219 (5.63 [4.52 to 6.74])	290 (6.67 [5.31 to 8.02])	312 (7.82 [6.79 to 8.86])	404 (9.22 [7.70 to 10.74])	396 (7.03 [6.43 to 7.63])	359 (6.15 [4.90 to 7.41])	325 (5.65 [4.60 to 6.70])	340 (6.40 [5.50 to 7.31])	491 (9.50 [8.37 to 10.64])	<.001	1.22 (1.03 to 1.45)	4.12 (2.12 to 6.87)
Ate diet products	108 (3.61 [2.32 to 4.91])	175 (4.65 [3.75 to 5.55])	191 (5.73 [4.77 to 6.68])	270 (7.03 [5.74 to 8.32])	247 (5.72 [4.35 to 7.09])	226 (4.42 [3.75 to 5.08])	161 (3.24 [2.06 to 4.42])	207 (4.12 [3.44 to 4.79])	236 (5.03 [4.15 to 5.91])	.31	1.46 (1.15 to 1.85)	2.10 (0.11 to 2.89)
Used liquid diet formula	95 (3.02 [2.16 to 3.88])	172 (4.01 [3.04 to 4.98])	125 (3.29 [2.66 to 3.92])	135 (3.45 [2.57 to 4.33])	96 (1.88 [1.47 to 2.28])	85 (1.45 [0.99 to 1.91])	87 (1.68 [1.02 to 2.34])	106 (2.27 [1.70 to 2.84])	132 (2.49 [1.75 to 3.23])	.76	0.80 (0.59 to 1.06)	−1.46 (−3.62 to 0.10)
Joined program to lose weight	32 (1.25 [0.45 to 2.06])	94 (2.71 [2.17 to 3.24])	116 (3.93 [3.25 to 4.62])	127 (3.81 [2.96 to 4.65])	116 (2.84 [2.05 to 3.63])	135 (2.90 [2.10 to 3.71])	118 (2.96 [2.13 to 3.80])	104 (2.68 [1.64 to 3.72])	90 (2.95 [1.99 to 3.92])	<.001	2.56 (1.71 to 3.80)	2.54 (0.45 to 3.76)
Took prescription diet pills	37 (0.92 [0.61 to 1.24])	50 (1.29 [0.83 to 1.75])	45 (1.34 [0.71 to 1.97])	50 (1.18 [0.77 to 1.58])	64 (1.18 [0.91 to 1.45])	52 (0.77 [0.43 to 1.12])	45 (0.97 [0.39 to 1.55])	75 (1.60 [1.16 to 2.04])	87 (1.43 [0.99 to 1.87])	.52	NA	NA
Took nonprescription supplement^d^	102 (3.14 [2.42 to 3.86)]	179 (4.73 [3.61 to 5.85])	117 (3.54 [2.54 to 4.54])	167 (4.45 [3.41 to 5.49])	142 (2.69 [2.13 to 3.26])	116 (2.15 [1.45 to 2.86])	112 (2.28 [1.66 to 2.90])	177 (3.44 [2.54 to 4.34])	182 (3.86 [3.07 to 4.66])	.001	NA	NA
Took laxatives	11 (0.31 [0.20 to 0.42])	39 (1.04 [0.48 to 1.59])	30 (0.73 [0.38 to 1.08])	19 (0.38 [0.09 to 0.67])	18 (0.28 [0.11 to 0.46])	22 (0.31 [0.13 to 0.49])	15 (0.19 [0.02 to 0.36])	22 (0.26 [0.13 to 0.39])	32 (0.44 [0.25 to 0.64])	<.001	NA	NA
Other methods	30 (0.72 [0.33 to 1.11])	55 (1.20 [0.65 to 1.74])	51 (1.24 [0.72 to 1.76])	44 (1.21 [0.67 to 1.75])	42 (0.73 [0.45 to 1.01])	27 (0.45 [0.19 to 0.70])	46 (0.91 [0.68 to 1.14])	25 (0.37 [0.14 to 0.61])	25 (0.43 [0.20 to 0.67])	.06	NA	NA
Drank a lot of water	7 (0.15 [0.00 to 0.30])	476 (12.26 [9.25 to 15.28])	576 (16.42 [14.73 to 18.10])	868 (21.59 [19.14 to 24.04])	913 (17.43 [15.14 to 19.72])	819 (14.38 [12.91 to 15.86])	767 (15.09 [13.62 to 16.55])	880 (17.17 [15.75 to 18.60])	1370 (26.30 [23.94 to 28.67])	<.001	5.16 (4.65 to 5.73)^e^	26.15 (24.13 to 28.98)
Followed a special diet	6 (0.15 [0 to 0.31])	109 (2.90 [1.59 to 4.21])	191 (6.03 [4.29 to 7.76])	199 (5.94 [5.03 to 6.86])	112 (2.52 [1.91 to 3.13])	102 (1.89 [1.51 to 2.27])	111 (2.50 [1.72 to 3.27])	158 (3.73 [3.03 to 4.42])	134 (3.34 [2.69 to 3.99])	<.001	NA	3.19 (0.61 to 3.78)
Ate fewer carbohydrates	NA	NA	NA	584 (15.67 [13.87 to 17.48])	632 (11.76 [9.41 to 14.10])	501 (8.94 [8.10 to 9.77])	489 (10.02 [8.58 to 11.45])	626 (12.93 [11.60 to 14.27])	755 (16.67 [14.59 to 18.75])	.03	1.03 (0.92 to 1.16)	1.00 (0.16 to 3.81)
Started or resumed smoking habit	NA	NA	NA	24 (0.68 [0.36 to 1.00])	15 (0.25 [0.06 to 0.44])	23 (0.34 [0.09 to 0.58])	17 (0.43 [0.09 to 0.78])	17 (0.39 [0.12 to 0.66])	41 (0.77 [0.47 to 1.07])	NA	NA	NA
Ate more fruits, vegetables, and/or salads	NA	NA	NA	6 (0.11 [0.00 to 0.24])	10 (0.19 [0.06 to 0.32])	881 (16.25 [14.92 to 17.59])	826 (16.54 [14.20 to 18.89])	974 (19.61 [18.55 to 20.66])	1450 (29.43 [26.99 to 31.87])	<.001	2.12 (1.93 to 2.33)^f^	30.32 (28.09 to 31.19)
Changed eating habits	NA	NA	NA	15 (0.32 [0.10 to 0.54])	15 (0. 35 [0.12 to 0.59])	604 (11.05 [9.60 to 12.50])	614 (12.35 [10.50 to 14.20])	883 (17.19 [15.90 to 18.47])	1036 (20.48 [18.06 to 22.89])	<.001	2.07 (1.86 to 2.31)^f^	20.16 (19.08 to 22.26)
Ate less sugar, candy, and/or sweets	NA	NA	NA	11 (0.23 [0.08 to 0.39])	29 (0.66 [0.34 to 0.97])	666 (11.99 [10.67 to 13.31])	559 (10.96 [9.81 to 12.12])	820 (16.64 [15.35 to 17.93])	1036 (20.94 [19.08 to 22.80])	<.001	1.87 (1.69 to 2.08)^f^	21.71 (19.26 to 22.59)
Ate less junk food or fast food	NA	NA	NA	NA	NA	695 (12.76 [11.49 to 14.04])	658 (13.18 [11.54 to 14.82])	851 (16.97 [15.82 to 18.13])	1178 (24.28 [22.53 to 26.03])	<.001	2.09 (1.88 to 2.31)^f^	12.09 (10.10 to 14.51)

Abbreviation: NA, not applicable.

^a^ Analyses were restricted to nonpregnant participants 20 years and older who responded to the National Health and Nutrition Examination Survey. Not all participants answered all questions; therefore, the number of participants in different categories were not equal to the total number of participants for the survey cycle. Increasing trends were defined as differences greater than 0 or a ratio greater than 1 with *P* for trend ≤ .001; decreasing trends were defined as differences less than 0 or a ratio of less than 1, with a *P* for trend ≤ .001; stable trends were defined as *P* for trend > .001.

^b^ Adjusted for age, sex, and race/ethnicity.

^c^ Calculated based on the available data of the relevant years.

^d^ Took pills, medicines, herbs, or supplements not needing a prescription.

^e^ Prevalence ratios for 2015-2016 vs 2001-2002 are presented.

^f^ Prevalence ratios for 2015-2016 vs 2009-2010 are presented.

Table 4 shows the cross-tabulated analyses of current measured BMI and self-perceived weight status. Among those who had overweight or obesity, a decreased trend was observed for the proportion of participants who considered themselves as having overweight, whereas an increased trend was observed for the proportion of participants who considered themselves as having underweight or about the right weight (difference, 3.3%; 95% CI, 1.0%-5.6%; *P *for trend < .001) (Table 4).

**Table 4.** Cross-Tabulated Type Analyses of Trends in Current Measured BMI and Self-perceived Weight Status Among Adults in the United States, 1999-2000 to 2015-2016^a^

BMI and Self-perceived Weight Status	1999-2000 (n = 4597)	2001-2002 (n = 5094)	2003-2004 (n = 4808)	2005-2006 (n = 4643)	2007-2008 (n = 5878)	2009-2010 (n = 6150)	2011-2012 (n = 5503)	2013-2014 (n = 5704)	2015-2016 (n = 5649)	*P* for Trend^b^	2015-2016 vs 1999-2000	
											Ratio (95% CI)	Difference, % (95% CI)
**Self-perception of Weight**												
Current measured BMI <25, No. (%)	1327 (28.87)	1430 (28.07)	1412 (29.37)	1333 (28.71)	1592 (27.08)	1667 (27.11)	1660 (30.17)	1643 (28.80)	1465 (25.93)	NA	NA	NA
Considered self as having overweight, No. (% [95% CI])	227 (20.18 [16.84 to 20.52])	236 (20.39 [17.85 to 23.91])	228 (20.64 [17.09 to 24.18])	220 (20.67 [16.92 to 24.42])	199 15.78 [13.73 to 17.83])	216 (14.68 [11.71 to 17.64])	184 (12.16 [9.26 to 15.05])	192 (11.52 [9.91 to 13.13])	132 (10.84 [8.34 to 13.34])	<.001	0.48 (0.38 to 0.60)	8.10 (5.63 to 10.57)
Considered self as having underweight or about the right weight, No. (% [95% CI])	1100 (79.82 [79.48 to 83.16])	1194 (79.61 [77.09 to 82.15])	1184 (79.36 [75.81 to 82.91])	1113 (79.33 ([75.58 to 83.08])	1393 (84.22 ([82.17 to 86.27])	1451 (85.32 ([82.36 to 88.29])	1476 (87.84 [84.95 to 90.74])	1451 (88.48 [86.87 to 90.09])	1333 (89.16 [86.66 to 91.66])			
Current measured BMI ≥25, No. (%)	2775 (60.37)	2972 (58.34)	3010 (62.60)	3017 (64.98)	3916 (66.62)	4242 (68.98)	3510 (63.78)	3801 (66.64)	3858 (68.30)	NA	NA	NA
Considered self as having overweight, No. (% [95% CI])	1956 (75.71 [73.44 to 77.97])	2133 (76.77 [74.76 to 78.74])	2146 (76.47 [74.56 to 78.38])	2185 (77.42 [74.98 to 80.02])	2762 (74.99 [72.56 to 77.33])	3005 (74.4 [72.45 to 76.35])	2401 (72.2 [68.66 to 75.73]	2702 (74.48 [72.86 to 76.10])	2592 (71.64 [69.30 to 73.98])	<.001	0.86 (0.77 to 0.95)	3.30 (1.04 to 5.57)
Considered self as having underweight or about the right weight, No. (% [95% CI])	819 (24.29 [22.03 to 26.56])	839 (23.25 [21.26 to 25.24])	864 (23.53 [21.62 to 25.44])	832 (22.5 [19.98 to 25.02])	1154 (25.01 [22.67 to 27.44])	1237 (25.6 [23.65 to 27.55])	1109 (27.8 [24.27 to 31.34])	1099 (25.52 [23.90 to 27.14])	1266 (28.36 [26.02 to 30.70])			
**Intention to Weigh More, the Same, or Less**												
Current, measured BMI <25, No. (%)	1328 (28.89)	1432 (28.11)	1412 (29.37)	1335 (28.75)	1594 (27.12)	1667 (27.11)	1659 (30.15)	1645 (28.84)	1466 (25.95)	NA	NA	NA
Intended to weigh more or the same amount, No. (% [95% CI])	1001 (68.74 [63.76 to 73.73])	1068 (67.40 [64.75 to 70.06])	1082 (69.86 [65.44 to 74.28])	981 (66.33 [61.74 to 70.93])	1219 (69.09 [65.30 to 72.90])	1247 (69.00 [65.67 to 72.35])	1282 (71.20 [65.90 to 76.52])	1266 (73.01 [68.88 to 77.32])	1137 (72.00 [67.42 to 76.70])	.03	1.13 (0.95 to 1.35)	2.18 (–0.97 to 5.33)
Intended to weigh less, No. (% [95% CI])	327 (31.26 [26.27 to 36.24])	364 (32.60 [29.94 to 35.25])	330 (30.14 [25.72 to 34.56])	354 (33.67 [29.07 to 38.26])	375 (30.91 [27.10 to 34.70])	420 (31.00 [27.65 to 34.33])	377 (28.80 [23.48 to 34.10])	379 (22.99 [22.68 to 31.12])	329 (28.00 [23.30 to 32.58])			
Current measured BMI ≥25. No. (%)	2778 (60.4)	2976 (58.42)	3012 (62.65)	3020 (65.04)	3921 (66.71)	4256 (69.20)	3516 (63.89)	3803 (66.67)	3869 (3869)	NA	NA	NA
Intended to weigh more or the same amount, No. (% [95% CI])	701 (19.00 [17.64 to 22.01])	709 (18.12 [16.21 to 20.04])	747 (19.40 [17.42 to 21.38])	701 (17.21 [15.36 to 19.07])	910 (17.85 [15.49 to 20.21])	994 (19.40 [17.72 to 21.09])	859 (19.54 [17.07 to 22.02])	836 (18.64 [17.16 to 20.13])	875 (18.23 [16.04 to 20.44])	.004	0.87 (0.77 to 0.97)	–2.62 (–4.69 to –0.55)
Intended to weigh less, No. (% [95% CI])	2077 (81.00 [77.99 to 82.36])	2267 (81.88 [79.96 to 83.79])	2265 (80.6 [78.62 to 82.58])	2319 (82.79 [80.93 to 84.64])	3011 (82.15 [79.79 to 84.51])	3262 (80.60 [78.91 to 82.28])	2657 (80.46 [77.98 to 82.93])	2967 (81.36 [79.87 to 82.84])	2994 (81.77 [79.56 to 83.96])			

Abbreviation: BMI, body mass index, calculated as weight in kilograms divided by height in meters squared.

^a^ Analyses were restricted to nonpregnant participants 20 years and older who responded to the National Health and Nutrition Examination Survey. Not all participants answered all questions; therefore, the number of participants in different categories were not equal to the total number of participants for the survey cycle. Increasing trends were defined as differences greater than 0 or a ratio greater than 1 with *P* for trend ≤ .001; decreasing trends were defined as differences less than 0 or a ratio of less than 1, with a *P* for trend ≤ .001; stable trends were defined as *P* for trend > .001.

^b^ Adjusted for age, sex, and race/ethnicity.

Table 5 presents the cross-tabulated analyses of current measured BMI with self-perceived weight status among US adults who pursued weight loss strategies. Among those with a BMI of at least 25 who considered themselves as having overweight, we observed increased trends for attempting to lose weight by eating less food (11.3%; 95% CI, 8.5%-14.1%; *P* < .001); exercising (18.1%; 95% CI, 15.4%-20.8%; *P* < .001), and drinking a lot of water (37.8%; 95% CI, 35.7%-40.0%; *P* < .001).

**Table 5.** Cross-Tabulated Type Analyses of Trends in Current Measured BMI, Self-perception of Weight, and Weight Loss Strategies Among Adults in the United States, 1999-2000 to 2015-2016^a^

Strategy	1999-2000 (n = 4597)	2001-2002 (n = 5094)	2003-2004 (n = 4808)	2005-2006 (n = 4643)	2007-2008 (n = 5878)	2009-2010 (n = 6150)	2011-2012 (n = 5503)	2013-2014 (n = 5704)	2015-2016 (n = 5649)	*P* for Trend^b^	2015-2016 vs 1999-2000	
											Ratio (95% CI)	Difference, % (95% CI)
BMI <25 and considered self as having overweight, No. (%)	227 (4.94)	236 (4.63)	228 (4.74)	220 (4.74)	199 (3.39)	216 (3.51)	184 (3.34)	192 (3.37)	132 (2.34)	NA	NA	NA
Ate less food, No. (% [95% CI])	55 (24.71 [17.31 to 32.12])	81 (31.66 [22.82 to 40.50])	83 (39.98 [30.64 to 49.31])	90 (44.27 [37.06 to 51.49])	64 (34.53 [24.37 to 44.70])	63 (34.38 [26.56 to 42.21])	55 (36.04 [24.98 to 47.10])	60 (34.10 [25.11 to 43.09])	45 (35.56 [25.76 to 45.36])	.84	1.62 (1.01 to 2.59)	9.86 (0.24 to 19.48)
Exercised, No. (% [95% CI])	64 (28.96 [21.17 to 36.74])	85 (34.20 [27.05 to 41.34])	76 (35.64 [28.31 to 42.99])	94 (46.41 [35.85 to 56.98])	66 (39.96 [29.23 to 50.68])	81 (41.41 [29.93 to 52.91])	55 (28.53 [18.78 to 38.29])	78 (43.17 [34.54 to 51.80])	50 (39.96 [30.80 to 49.12])	.84	1.62 (1.01 to 2.59)	9.86 (0.24 to 19.48)
Drank a lot of water, No. (% [95% CI])	0	32 (12.95 [6.20 to 19.69])	38 (18.73 [13.93 to 23.52])	63 (31.78 [21.29 to 42.27])	39 (20.93 [12.67 to 29.19])	34 (16.79 [12.58 to 20.99])	55 (17.62 [10.84 to 24.42])	9 (20.01 [12.02 to 28.01])	37 (30.34 [16.98 to 43.71])	<.001	>999.99 (<0.01 to >999.99)	28.03 (22.15 to 33.91)
Ate more fruits, vegetables, and/or salads, No. (% [95% CI])	NA	NA	NA	0	1 (0.50 [–0.25 to 0.69])	49 (27.01 ([21.53 to 32.50])	55 (25.7 [15.25 to 36.14])	40 (21.01 [13.35 to 28.68])	37 (34.1 [21.16 to 47.06])	<.001	>999.99 (<0.01 to >999.99)	28.03 (22.06 to 34.00)
Changed eating habits, No. (% [95% CI])	NA	NA	NA	1 (0.56 [–0.58 to 1.71])	1 (0.32 [–0.37 to 1.00])	34 (14.56 [10.64 to 18.48])	39 (17.40 [9.37 to 25.44])	41 (21.64 [13.40 to 29.88])	28 (18.65 [10.12 to 27.18])	<.001	58.96 (7.91 to 439.28)	20.76 (15.20 to 26.32)
Ate less sugar, candy, or sweets, No. (% [95% CI])	NA	NA	NA	1 (0.17 [–0.19 to 0.52])	1 (0.22 [–0.25 to 0.69])	29 (15.58 [8.65 to 22.50])	30 (16.12 [9.08 to 23.15])	38 (22.29 [14.62 to 29.96])	28 (23.45 [12.15 to 34.74])	<.001	58.96 (7.91 to 439.28)	20.76 (15.20 to 26.32)
BMI <25 and considered self as having underweight or about the right weight, No. (%)	1100 (23.93)	1194 (23.44)	1184 (24.63)	1113 (23.97)	1393 (23.70)	1451 (23.60)	1476 (26.82)	1451 (25.44)	1333 (23.60)	NA	NA	NA
Ate less food, No. (% [95% CI])	52 (6.92 [4.17 to 9.59])	87 (9.10 [6.23 to 12.01])	90 (10.00 [7.32 to 12.76])	104 (11.22 [8.54 to 13.91])	108 (9.94 [7.64 to 12.24])	107 (9.36 [7.64 to 11.09])	118 (12.05 [7.82 to 16.29])	121 (11.84 [9.54 to 14.21])	136 (13.69 [10.16 to 17.24])	<.001	2.29 (1.65 to 3.19)	5.48 (3.35 to 7.60)
Exercised, No. (% [95% CI])	51 (4.64 [4.42 to 9.73])	81 (10.07 [7.31 to 12.85])	92 (9.76 [6.95 to 12.57])	105 (12.29 [8.97 to 15.61])	125 (12.89 [10.16 to 15.60])	140 (12.88 [10.91 to 14.84])	160 (14.03 [10.42 to 17.66])	169 (15.82 [12.75 to 18.91])	177 (15.48 [11.98 to 18.98])	<.001	3.15 (2.28 to 4.35)	8.64 (6.34 to 10.95)
Drank a lot of water, No. (% [95% CI])	1 (0.12 [–0.14 to 0.38])	33 (3.63 [1.54 to 5.72])	48 (5.67 [4.02 to 7.32])	58 (6.10 [3.63 to 8.57])	62 (6.34 [4.26 to 8.42])	67 (5.79 [4.17 to 7.41])	118 (5.65 [3.83 to 7.47])	34 (8.05 [6.29 to 9.82])	113 (9.74 [7.50 to 11.98])	<.001	101.79 (14.19 to 730.13)	8.39 (6.73 to 10.04)
Ate more fruits, vegetables, and/or salads, No. (% [95% CI])	NA	NA	NA	1 (0.12 [–0.14 to 0.39])	0	85 (8.80 [6.64 to 10.96])	160 (8.07 [5.76 to 10.38])	101 (10.20 [8.24 to 12.18])	148 (14.25 [11.22 to 17.19])	<.001	138.88 (19.40 to 994.05)	11.01 (9.16 to 12.87)
Changed eating habits, No. (% [95% CI])	NA	NA	NA	0	2 (0.27 [–0.12 to 1.22])	64 (6.35 [3.95 to 6.59])	90 (4.87 [3.07 to 7.59])	86 (7.89 [6.01 to 10.28])	96 (9.14 [6.14 to 12.23])	<.001	28.68 (9.07 to 90.71)	6.93 (5.39 to 8.48)
Ate less sugar, candy, or sweets, No. (% [95% CI])	NA	NA	NA	3 (0.34 [–0.01 to 0.69])	2 (0.27 [–0.14 to 0.68])	64 (6.35 [4.73 to 7.99])	90 (4.87 [3.35 to 6.39])	86 (7.89 [5.92 to 9.85])	96 (9.14 [6.20 to 12.10])	<.001	>999.99 (<0.01 to >999.99)	7.20 (5.68 to 8.72)
BMI ≥25 and considered self as having overweight, No. (%)	1956 (42.55)	2133 (41.87)	2146 (44.63)	2185 (47.06)	2762 (46.99)	3005 (48.86)	2401 (43.63)	2702 (47.37)	2592 (45.88)	NA	NA	NA
Ate less food, No. (% [95% CI])	628 (33.43 [29.57 to 37.29])	812 (38.92 [36.62 to 41.22])	835 (39.34 [36.56 to 42.14])	928 (42.70 [40.90 to 44.50])	1180 (43.24 [40.51 to 45.98])	1024 (34.60 [31.10 to 38.10])	948 (39.80 [36.48 to 43.11])	1102 (41.15 [37.97 to 44.34])	1125 (45.56 [42.58 to 48.62])	<.001	1.62 (1.43 to 1.83)	11.30 (8.46 to 14.14)
Exercised, No. (% [95% CI])	447 (25.91 [23.79 to 28.03])	682 (34.55 [31.55 to 37.55])	658 (33.75 [30.81 to 36.71])	829 (39.97 [36.50 to 43.44])	954 (37.19 [33.41 to 40.97])	974 (34.68 [31.97 to 37.40])	911 (37.54 [34.41 to 40.67])	1050 (38.85 [36.61 to 41.09])	1062 (42.12 [37.79 to 46.46])	<.001	2.34 (2.06 to 2.67)	18.12 (15.40 to 20.83)
Drank a lot of water, No. (% [95% CI])	5 (0.22 [–0.05 to 0.50])	352 (18.53 [14.05 to 23.01])	432 (24.45 [21.44 to 27.50])	658 (31.15 [27.95 to 34.35])	700 (25.67 [22.27 to 29.07])	617 (20.67 [17.78 to 23.56])	948 (21.72 [19.73 to 23.71])	220 (23.74 [21.49 to 25.99])	987 37.45 [34.30 to 40.61])	<.001	239.92 (99.40 to 579.10)	37.82 (35.66 to 39.98)
Ate more fruits, vegetables, and/or salads, No. (% [95% CI])	NA	NA	NA	5 (0.16 [0.01 to 0.31])	8 (0.18 [–0.031 to 0.40])	647 (22.15 [19.38 to 24.92])	911 (23.23 [20.53 to 25.94])	716 (27.10 [24.45 to 29.76])	1018 (40.89 [37.78 to 44.00])	<.001	281.92 (116.82 to 680.33)	39.05 (36.99 to 41.10)
Changed eating habits, No. (% [95% CI])	NA	NA	NA	4 (0.54 [0.20 to 0.88])	23 (0.29 [–0.07 to 0.65])	486 (15.88 [13.51 to 18.24])	579 (17.16 [15.09 to 19.24])	593 (23.42 [21.22 to 25.63])	750 (29.00 [25.78 to 32.21])	<.001	221.97 (82.94 to 594.06)	28.75 (26.84 to 30.66)
Ate less sugar, candy, or sweets, No. (% [95% CI])	NA	NA	NA	13 (0.18 [–0.02 to 0.38])	8 (1.09 [0.48 to 1.71])	448 (12.38 [11.10 to 14.05])	546 (15.46 [13.54 to 17.37])	638 (23.15 [21.04 to 25.26])	740 (29.74 [26.55 to 32.92])	<.001	66.76 (38.44 to 115.92)	27.95 (26.04 to 29.87)
BMI ≥25 and considered self as having underweight or about the right weight	819 (17.81)	839 (16.47)	864 (17.97)	832 (19.63)	1154 (19.63)	1237 (20.11)	1109 (20.15)	1099 (19.27)	1266 (22.41)	NA	NA	NA
Ate less food, No. (% [95% CI])	72 (9.26 [5.61 to 12.92])	120 (15.58 [11.52 to 19.63])	118 (16.05 [11.79 to 20.32])	132 (17.28 [13.65 to 21.12])	165 (16.36 [13.24 to 19.49])	181 (14.73 [13.38 to 16.09])	183 (17.90 [16.00 to 19.81])	195 (19.43 [16.84 to 22.02])	250 (19.84 [16.79 to 22.89])	<.001	2.55 (1.93 to 3.37)	10.96 (7.81 to 14.10)
Exercised, No. (% [95% CI])	65 (11.16 [6.28 to 16.04])	118 (17.84 [12.23 to 23.45])	108 (17.04 [13.61 to 20.48])	112 (16.74 [12.95 to 20.52])	154 (16.28 [12.67 to 19.90])	189 (16.46 [14.01 to 18.92])	212 (21.84 [17.36 to 26.33])	223 (22.34 [19.16 to 25.53])	299 (24.43 [20.91 to 27.78])	<.001	3.59 (2.70 to 4.77)	15.68 (12.41 to 18.95)
Drank a lot of water, No. (% [95% CI])	1 (0.60 [–0.07 to 1.20])	39 (7.47 [3.60 to 11.35])	51 (8.71 [6.63 to 10.80])	79 (11.30 [9.60 to 13.00])	105 (10.74 [7.96 to 13.53])	97 (8.64 [6.83 to 10.47])	183 (11.22 [8.80 to 13.65])	33 (11.70 [9.14 to 14.26])	218 (18.98 [15.16 to 22.80])	<.001	170.12 (23.81 to >999.99)	17.10 (14.50 to 19.69)
Ate more fruits, vegetables, and/or salads, No. (% [95% CI])	NA	NA	NA	0	3 (0.47 [–0.15 to 1.08])	96 (8.08 [6.21 to 9.96])	212 (9.82 [6.50 to 13.14])	111 (11.91 [9.17 to 14.65])	236 (19.90 [15.60 to 24.20])	<.001	>999.99 (<0.01 to >999.99)	18.64 (15.99 to 21.29)
Changed eating habits, No. (% [95% CI])	NA	NA	NA	1 (0.09 [–0.10 to 0.28])	3 (0.33 [–0.05 to 0.70])	79 (5.35 [3.34 to 7.36])	106 (9.20 [5.46 to 12.93])	95 (12.00 [8.85 to 15.16])	151 (13.86 [10.17 to 17.54])	<.001	112.54 (15.72 to 805.73)	11.81 (9.59 to 14.02)
Ate less sugar, candy, or sweets, No. (% [95% CI])	NA	NA	NA	NA	1 (0.10 [–0.11 to 0.31])	3 (0.19 [–0.08 to 0.47])	64 (7.37 [5.27 to 9.47])	113 (7.41 [4.17 to 10.66])	113 (9.92 [7.55 to 12.30])	<.001	119.35 (16.68 to 854.26)	12.44 (10.18 to 14.70)

Abbreviation: BMI, body mass index (calculated as weight in kilograms divided by height in meters squared); NA, not applicable.

^a^ Analyses were restricted to nonpregnant participants 20 years and older who responded to the National Health and Nutrition Examination Survey. Not all participants answered all questions; therefore, the number of participants in different categories were not equal to the total number of participants for the survey cycle. Increasing trends were defined as differences greater than 0 or a ratio greater than 1 with *P* for trend ≤ .001; decreasing trends were defined as differences less than 0 or a ratio of less than 1, with a *P* for trend ≤ .001; stable trends were defined as *P* for trend > .001.

^b^ Adjusted for age, sex, and race/ethnicity.

## Discussion

This analysis of nationally representative data collected from US adults during NHANES 1999-2000 through NHANES 2015-2016 revealed increasing trends in current measured BMI and weight; adjusted, self-reported, prior-year weight; and the difference between current measured weight and adjusted, self-reported, prior-year weight. The prevalence of participants who tried to lose weight in the past year also increased over time. Overall, reduced food consumption, exercise, and consumption of a large volume of water were the most frequently applied weight loss strategies. In addition, the proportion of participants who reported consuming more fruits, vegetables, and salads, changing their eating habits, consuming less sugar, candy, and sweets, or consuming less junk food or fast food increased sharply over time and became the most commonly applied weight loss strategies in recent years.

From 2007-2008 to 2015-2016, the prevalence of obesity among US adults increased from 33.7% to 39.6%.^1^ We also observed increasing trends in actually measured BMI and weight and self-perceived weight status from 1999-2000 to 2015-2016 in parallel with the increasing proportion of participants who tried to lose weight.

Unsurprisingly, among those who had overweight and obesity and pursued weight loss strategies, we observed an increased trend in the proportion of participants who considered themselves as having overweight. However, evidence suggests that a self-perception of having overweight is not reliably associated with physical activity or healthy eating.^5^ In addition, much evidence has implied that self-perceived overweight was associated with increased weight gain over time.^5^

Additionally, a previous study emphasized that less than 34% of the US population reported attempted weight loss in response to an inquiry.^13^ Social pressure associated with an acceptable body weight and size might contribute to the increased reporting of weight loss attempts.^14^ Despite the weighted prevalence of participants who attempted to lose weight in the past year increasing from 34.3% to 42.2% during the study period, we observed increased trends for current measured BMI and weight and the difference between current measured weight and self-reported, prior-year weight among those had attempted to lose weight in the past year. Taken together, these findings suggest that although 34.3% to 42.2% of adults in the United States in our study reported weight loss efforts, many of them might not have actually implemented weight loss strategies or applied a minimal level of effort, which yielded unsatisfactory results.

Although reduced food consumption was among the most commonly reported strategies by participants who attempted to lose weight, no significant trends were observed for the proportion of participants who reported lowering calories, implying that energy intake was not decreased. Furthermore, reduced food consumption might be a general strategy applied by adults in response to social pressure, despite evidence supporting the association of reduced food (ie, calorie) consumption and significant weight loss.^15,16^ However, adherence to such modified diets, which is the best predictor of success in any dietary modification, is very difficult to maintain.^17^ Exercising was another of the most commonly reported strategies by participants who attempted to lose weight; however, a 2008 study^18^ reported that while 65% of adults in the United States reported that they met the recommended levels of physical activity, only 5% actually met these goals as objectively measured using accelerometry devices. Furthermore, a 2018 statement from the American Heart Association^19^ indicated that 8 in 10 adults in the United States did not satisfy the guidelines for aerobic and muscle-strengthening exercise. Therefore, decreased energy intake, adherence to reduced food consumption, and the quality of exercise are significant challenges to effective weight loss. An increasing trend in the use of reduced food consumption as a weight loss strategy was evidenced in the proportion of participants in our study; however, no significant change over time was observed in the proportion of overall participants who reported lowering calories or reducing carbohydrate consumption to lose weight.

Our analysis revealed an increasing trend in the use of increased water consumption as a weight loss strategy. This increase can be attributed to convincing evidence regarding the potentially important role of water in reducing energy intake, which thus contributes to the long-term maintenance of weight loss.^20^

From NHANES 2005-2006 to NHANES 2015-2016, the proportion of participants who reported consuming more fruits, vegetables, and salads, changing their eating habits, consuming less sugar, candy, and sweets, and consuming less junk food or fast food increased sharply. The sharp increase might be partly because these categories were added to NHANES in the 2005-2006 cycle. However, as the trends for actually measured weight and self-reported weight history increased during the same time period, these strategies may not have translated into effective weight loss.^5^ Evidence from other studies^21^ has shown these changes to be associated with less weight gain, and therefore, these strategies would be encouraged.

Only slight changes were observed in reduced fat consumption in participants during the study period; however, among adults in the United States with obesity who participated in the NHANES between 2001 and 2006, a large proportion were more likely to report a body weight loss of 10% or more in response to reduced fat consumption, increased exercise, the use of prescription weight loss medications, or participation in a commercial weight loss program.^7^ Existing guidelines and compelling evidence also suggest that longitudinal weight management relies on a combination of reductions in energy and fat intake, an increase in dietary fiber intake, regular physical activity, self-monitoring, and other behavioral techniques.^22^ Reduction of either carbohydrates or fat has been similarly related to weight loss, especially in the context of low-calorie diets.^23^

It is worth noting that specific dietary or lifestyle factors may independently improve weight loss and need to be targeted.^21^ Taken together, these findings suggest a need to increase the promotion of effective strategies for weight loss, including caloric reduction and increased physical activity, among all adults attempting to lose weight.^24^ Notably, adherence is the primary factor associated with a successful response to a weight loss attempt.^25^ Therefore, weight loss strategies that consider a participant’s preferences and abilities may help to optimize participant adherence.^26^

In fact, those who attempted weight loss might not be the participants who truly needed to lose weight, and others might need to lose weight but did not attempt to do so because they perceived their weight as approximately the right weight. In our study, a decreased trend was observed for the proportion of participants with overweight or obesity who considered themselves as having overweight. Among those who attempted to lose weight, we observed an increased trend for the difference between current measured weight and self-reported, prior-year weight.

### Strengths and Limitations

The identified trends in current measured BMI and weight, self-perceived weight status, and weight loss attempts and strategies were determined using nationally representative data. Therefore, the results may be generalizable to other adults in the United States. In our analyses, Bonferroni correction was used to reduce the type I error. However, several potential limitations of our study should be acknowledged. First, the self-perceived weight status and weight loss attempts and strategies were based on self-reported data. However, according to our data, current self-reported weight was only slightly lower than measured weight (difference, 1.19-2.09 pounds), and the trends of these 2 measures were similar. In addition, previous studies reported a correlation of at least 90% between self-reported and actual weights,^27^ suggesting that the recall of weight history is relatively stable and subject to minimal bias.^28^ Further, we applied the corrected equation to calculate the adjusted, self-reported, prior-year weight.^8^ Second, the NHANES did not collect data on the frequency, duration, or number of weight loss attempts or strategies. Repeated weight loss attempts have been shown to reduce participants’ beliefs in the long-term effects of weight loss efforts.^29^ Third, temporal relationships and causality could not be established because of the cross-sectional design of NHANES.

## Conclusions

In conclusion, our analysis of nationally representative data collected from adults in the United States who participated in the NHANES from 1999-2000 to 2015-2016 revealed increasing trends in actually measured BMI and weight; adjusted, self-reported, prior-year weight; and the difference between current measured weight and adjusted, self-reported, prior-year weight. These increases were observed despite increases in the proportion of participants who attempted to lose weight and used weight loss strategies, such as reducing food consumption, exercising, and consuming a large volume of water.

## Notes

Supplement. **eFigure.** Flow Chart of Sample Selection **eTable 1.** Baseline Characteristics of Adults in the United States from NHANES, 1999-2000 to 2015-2016 **eTable 2.** Trends in Self-reported Weight Among Adults in the United States, 1999-2000 to 2015-2016
