# Error in Title and Table

**DOI:** 10.1001/jamanetworkopen.2019.19047

**Published:** 2019-12-06

In the Original Investigation titled “Trends in Self-perceived Weight Status, Weight Loss Attempts, and Weight Loss Strategies Among Adults in the United States, 1999-2016,”^1^ published November 13, 2019, there were errors in the title and in Table 3. The title should have appeared as “Trends in Self-perceived Weight Status, Weight Loss Attempts, and Weight Loss Strategies Among Adults in the United States, 1999-2016,” and the second column heading in Table 3 should have appeared as “1999-2000.” This article has been corrected.^1^
