# Supplementary material for: Trends in Self-perceived Weight Status, Weight Loss Attempts, and Weight Loss Strategies Among Adults in the United States, 1999-2006
Source: JAMA Netw Open. 2019 Nov 13;2(11):e1915219. doi: 10.1001/jamanetworkopen.2019.15219 (PMC6902793; doi:10.1001/jamanetworkopen.2019.15219)
Supplement: Supplement. — eFigure. Flow Chart of Sample Selection eTable 1. Baseline Characteristics of Adults in the United States from NHANES, 1999-2000 to 2015-2016 eTable 2. Trends in Self-reported Weight Among Adults in the United States, 1999-2000 to 2015-2016 [file jamanetwopen-2-e1915219-s001.pdf]

Supplementary Online Content

Han L, You D, Zeng F, et al. Trends in self-perceived weight status, weight loss attempts, and weight loss strategies among adults in the United States, 1999-2006. *JAMA Netw Open.* 2019;2(11): e1915219. doi:10.1001/jamanetworkopen.2019.15219

eFigure. Flow Chart of Sample Selection
eTable 1. Baseline Characteristics of Adults in the United States from NHANES, 1999-2000 to 2015-2016
eTable 2. Trends in Self-reported Weight Among Adults in the United States, 1999-2000 to 2015-2016

This supplementary material has been provided by the authors to give readers additional information about their work.

© 2019 Han L et al. *JAMA Network Open.*

eFigure. Flow Chart of Sample Selection

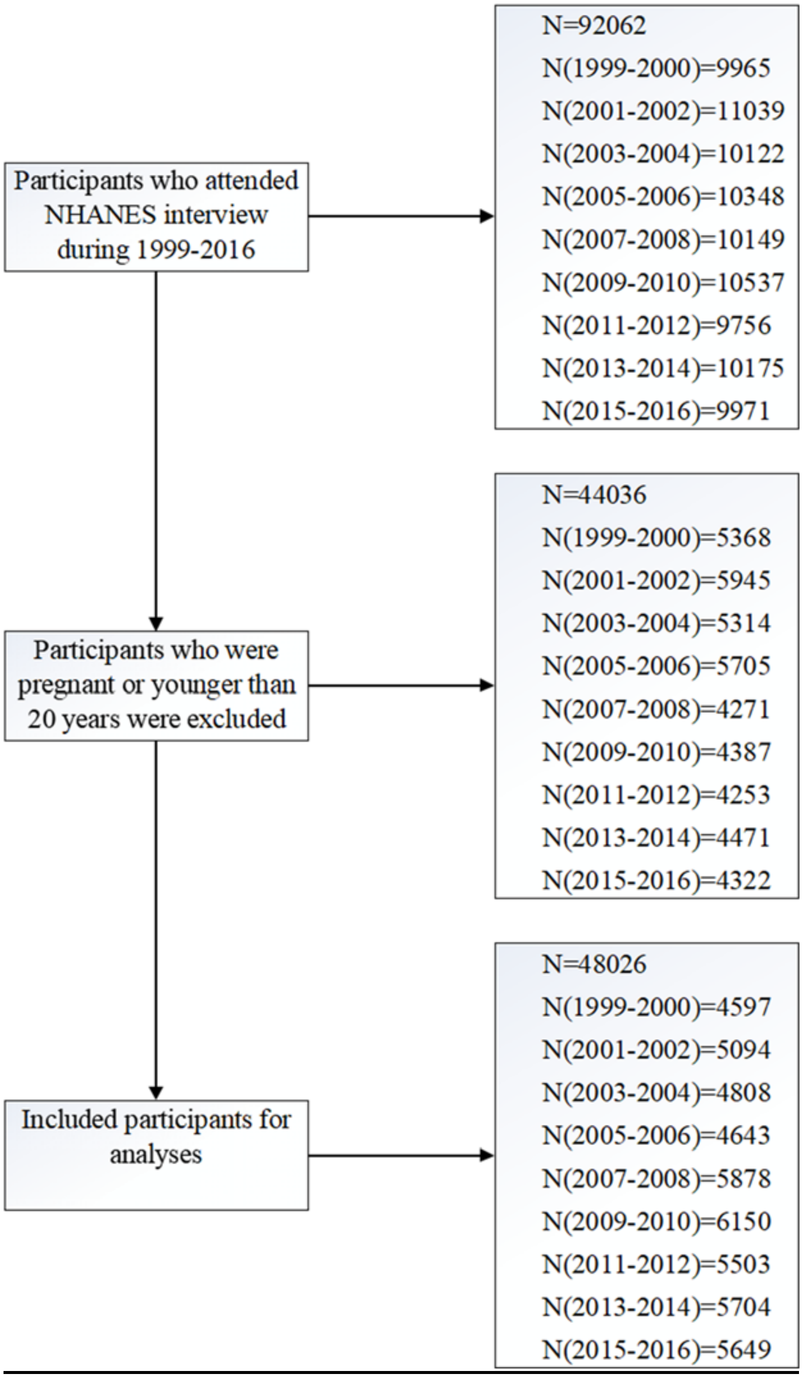

© 2019 Han L et al. *JAMA Network Open.*

eTable 1. Baseline Characteristics of Adults in the United States from NHANES 1999-2000 to 2015-2016

		1999-2000	2001-2002	2003-2004	2005-2006	2007-2008	2009-2010	2011-2012	2013-2014	2015-2016
Overall	N	4597	5094	4808	4643	5878	6150	5503	5704	5649
Age (years)	20-39	1419	1609	1511	1590	1855	2017	1903	1892	1887
	40-64	1786	2022	1803	1864	2467	2610	2350	2506	2384
	≥65	1392	1463	1494	1189	1556	1523	1250	1306	1378
Sex	Men	2269	2536	2418	2387	2910	3006	2740	2758	2747
	Women	2328	2558	2390	2256	2968	3144	2763	2946	2902
Race/Ethnicity	Non-Hispanic white	2085	2699	2573	2345	2752	2953	2023	2449	1846
	Non-Hispanic black	879	972	956	1077	1214	1109	1437	1157	1182
	Non-Hispanic Asian	1200	1038	923	897	1011	1120	534	763	982
	others	433	385	356	324	901	968	1509	1335	1639
BMI(kg/m^2)	18-25 normal	1237	1322	1320	1229	1475	1547	1526	1520	1356
	25-30 overweight	1430	1596	1537	1457	1880	1971	1635	1721	1687
	≥30 obese	1328	1340	1451	1532	2028	2255	1855	2060	2159

NHANES: National Health and Nutrition Examination Survey

We restricted our analyses to non-pregnant participants aged ⩾20 years. Not all the participants included in our study have answered all of the questions, therefore the numbers of participants in different categories were not equal to the total number of participant for the certain survey cycle.

© 2019 Han L et al. *JAMA Network Open.*

eTable 2. Trends in Self-reported Weight Among Adults in the United States, 1999-2000 to 2015-2016[a,b]

		1999-2000 (n=4597)	2001-2002 (n=5094)	2003-2004 (n=4808)	2005-2006 (n=4643)	2007-2008 (n=5878)	2009-2010 (n=6150)	2011-2012 (n=5503)	2013-2014 (n=5704)	2015-2016 (n=5649)	P Value for Trend [c]	2015-2016 vs 1999-2000 Difference [c]
Current self-reported Weight (pounds)	N	4099	4676	4461	4354	5539	5874	5184	5448	5180		
		174.59 (171.87-177.30)	175.40 (173.04-177.77)	177.18 (174.94-179.42)	178.90 (175.82-181.99)	178.49 (176.23-180.75)	179.01 (177.08-180.94)	178.64 (176.36-180.92)	180.94 (179.10-182.79)	182.55 (179.39-185.71)	<0.001	7.96 (4.59-9.15)
Self-reported weight-10 yrs ago (pounds)	N	3027	3982	3277	3121	4057	4203	3628	3962	3732		
		163.53 (160.96-166.10)	165.44 (162.86-168.02)	167.24 (165.20-169.28)	169.64 (167.41-171.87)	169.55 (167.08-172.03)	171.63 (170.32-172.93)	172.53 (169.83-175.23)	175.56 (173.30-177.83)	176.32 (172.96-179.69)	<0.001	12.79 (9.69-14.65)
Self-reported weight - age 25 (pounds)	N	3550	3982	3838	3728	4749	4941	4288	4639	4474		
		150.01 (147.68-152.33)	150.36 (148.81-151.92)	151.89 (150.09-153.70)	153.28 (151.05-155.50)	152.85 (151.58-154.12)	153.84 (152.57-155.12)	154.62 (152.94-156.30)	157.06 (155.28-158.85)	156.98 (154.80-159.17)	<0.001	6.97 (6.21-10.06)
Self-reported height - age 25 (inches)	N	2032	2215	2238	2039	2788	2790	2434	2606	2493		
		66.55 (66.30-66.80)	66.73 (66.58-66.88)	66.91 (66.67-67.15)	66.86 (66.65-67.08)	66.92 (66.66-67.19)	66.97 (66.80-67.13)	66.85 (66.58-67.12)	66.92 (66.63-67.21)	66.93 (66.59-67.27)	0.01	0.38 (0.13-0.60)

© 2019 Han L et al. *JAMA Network Open.*

Self-reported greatest weight (pounds)	N	4067	4650	4394	4274	5516	5881	5165	5444	5177		
		187.24 (184.16-190.33)	189.14 (187.07-191.20)	191.38 (188.86-193.89)	194.13 (190.57-197.70)	193.51 (190.48-196.54)	194.13 (192.17-196.08)	194.78 (191.79-197.78)	198.75 (196.45-201.04)	200.35 (196.58-204.11)	<0.001	13.11 (9.04-14.18)
Age when heaviest weight	N	4040	4624	4442	4349	5473	5843	5122	5410	5134		
		38.93 (38.19-39.67)	39.04 (38.08-40.00)	39.95 (39.06-40.85)	39.63 (38.37-40.89)	40.13 (39.53-40.73)	40.27 (39.32-41.23)	40.38 (38.95-41.81)	40.31 (39.69-40.94)	40.62 (39.54-41.70)	0.03	1.69 (1.06-1.91)

[a] Among adults 20 years and older.

[b] Increasing trends were defined as differences greater than 0 or a ratio greater than 1 with P for trend ≤.001; decreasing trends were defined as differences less than 0 or a ratio of less than 1, with a P for trend ≤.001; stable trends were defined as P for trend > .001.

[c] Adjusted for age, sex, and race/ethnicity.

© 2019 Han L et al. *JAMA Network Open.*
